# Income related inequality and influencing factors: a study for the incidence of catastrophic health expenditure in rural China

**DOI:** 10.1186/s12889-017-4713-x

**Published:** 2017-09-20

**Authors:** Hai Gu, Yun Kou, Zhiwen Yan, Yilei Ding, Jusheng Shieh, Jun Sun, Nan Cui, Qianjing Wang, Hua You

**Affiliations:** 1 Center for Health Policy and Management Studies, Nanjing University, Nanjing, 210093 China; 2 School of Basic Medical Sciences, Nanjing Medical University, Nanjing, 211166 China; 3 Division of Cooperative Medical Scheme, Department of Primary Health, National Health and Family Planning Commission of People’s Republic of China, Beijing, 100044 China; 4 Department of Social Medicine and Health Education, School of Public Health, Nanjing Medical University, Nanjing, 211166 China

**Keywords:** Catastrophic health care expenditure, Income related inequality, Concentration index, New Rural Cooperative Medical Scheme,Reimbursement, Out-of-pocket expenditures

## Abstract

**Background:**

Catastrophic health expenditure (CHE) puts a heavy disease burden on patients’ families, aggravating income-related inequality. In an attempt to reduce the financial risks of rural families incurring CHE, China began the New Rural Cooperative Medical System (NCMS) on a trial basis in 2003 and has raised the reimbursement rates continuously since then. Based on statistical data about rural families in sample area of Jiangsu province, this study measures the incidence of CHE, analyzes socioeconomic inequality related to CHE, and explores the influences of the NCMS on the incidence of CHE.

**Methods:**

Statistical data were acquired from two surveys about rural health care, one conducted in 2009 and one conducted in 2010. In 2009, 1424 rural families were analyzed; in 2010, 1796 rural families were analyzed. An index of CHE is created to enable the evaluation of the associated financial risks. The concentration index and concentration curve are used to measure the income-related inequality involved in CHE. Multiple logistic regression is utilized to explore the factors that influence the incidence of CHE.

**Results:**

The incidence of CHE decreased from 13.62% in 2009 to 7.74% in 2010. The concentration index of CHE was changed from −0.298 (2009) to −0.323 (2010). Compared with rural families in which all members were covered by the NCMS, rural families in which some members were not covered by the NCMS had a lower incidence of CHE: The odds ratio is 0.65 with a 95% confidence interval of 0.43 to 1.00. For rural families in which all members were covered by the NCMS, the increase in reimbursement rates is correlated to the decline in the incidence of CHE if other influencing factors were controlled: The odds ratio is 0.48 with a 95% confidence interval of 0.36 to 0.64.

**Conclusions:**

Between 2009 and 2010, the incidence rate of CHE in the sampled area decreased sharply, CHE was more concentrated among least wealthy and inequality increased during study period. As of 2010, the poorest rural families still had high risk of experiencing CHE. For rural families in which all members are covered by the NCMS, the rise in reimbursement rates reduces the probability of experiencing CHE.

**Electronic supplementary material:**

The online version of this article (10.1186/s12889-017-4713-x) contains supplementary material, which is available to authorized users.

## Background

In rural China, major diseases may carry financial risk to the patients’ families, and disease-related poverty has occurred from time to time [1]. Approximately 42% of China’s total poverty-stricken population of 70,000,000 live in poverty as a result of disease; in the eastern region of China, the disease-related poverty rate is even higher (60%) [2]. High medical expenses force patients’ families to cut expenditures on education, clothes, entertainment, and even subsistence items such as food, and such cuts may have catastrophic effects on the families’ present and future life [3]. Families in such circumstances have experienced catastrophic health expenditure (CHE). Given that the financial risks of health care are related to household income, the risks of experiencing CHE vary from family to family according to income level, thus magnifying the inequality. The World Health Organization noted that health care equality aims to reduce the inequality and undue social gap related to use of health care [4]. Most countries around the world have basic medical insurance systems, and the redistributive design of those systems is intended to reduce the socioeconomic inequality in CHE and improve health care equality [5].

In an attempt to reduce rural families’ financial burdens of high medical expenses and improve health care equality, China began the New Rural Cooperative Medical System (NCMS) on a trial basis in 2003 [6]. As a basic medical insurance system for China, the NCMS reimburses covered rural residents for a certain portion of their medical expenses, to reduce the financial burden of health care, improves their use of medical services, and is intended to improve their health [7]. The NCMS’s reimbursement rates have increased continuously since then [8]. Between 2009 and 2010, the NCMS’s reimbursement rates in the sample areas (Feng county and Tongshan county in Xuzhou, Jiangsu province) were adjusted as follows: the reimbursement rate for outpatient expenses were raised from 25 to 30%; the reimbursement rate for inpatient expenses were raised by 20%, from 60 to 80%.

Researches on the relationship between medical insurance and disease-related financial risk currently focused mainly on the correlation between medical insurance and the incidence of CHE. Some studies have analyzed changes in the incidence of CHE subsequent to the reimbursement of medical expenses, thus proving that medical insurance plays a role in lowering the financial risks of disease [1]. Such research also has shown that increasing reimbursement rates reduces the incidence of CHE [9]. According to National Health Service Survey (NHSS) statistics for Shaanxi Province in 2013, Xu found that the NCMS significantly reduced the incidence of CHE [10].

Medical insurance is actually a type of price subsidy, and the decline in medical costs will increase the people’s demand for medical services. Therefore, medical insurance will not necessarily reduce the incidence of CHE. Recent research findings have shown that medical insurance can reduce the incidence of CHE to an extent that varies depending on the group of people. While researching older people, Cheng found that the NCMS affected neither the out-of-pocket (OOP) expenditure nor the incidence of CHE [11]. Likewise, Zhou found that medical insurance could play only a limited role in reducing the incidence of CHE for chronic-disease patients and aged families but could reduce the incidence of CHE for low-income families [12]. Fang found that the NCMS did not effectively reduce the medical expenses of rural residents, especially poor rural residents [13]. Wagstaff found that the beneficiaries of medical insurance in China tended to use medical services and see doctors at high-level medical institutions more frequently than did the uninsured, unexpectedly raising the incidence of CHE [14].

Therefore, whether the NCMS—and adjustment of its reimbursement rates—can play an active role in reducing the incidence of CHE and bringing about social equality in health care mains to be seen. More evidence is required.

Incorporating the related statistical data about rural families of Jiangsu province as of 2009 and 2010, this study measures the incidence of CHE, analyzes the income-related inequality in CHE, and explores the role that the NCMS and adjustment of its reimbursement rates play in reducing rural residents’ incidence of CHE.

## Methods

### Data collection and study samples

**Fig. 1:**
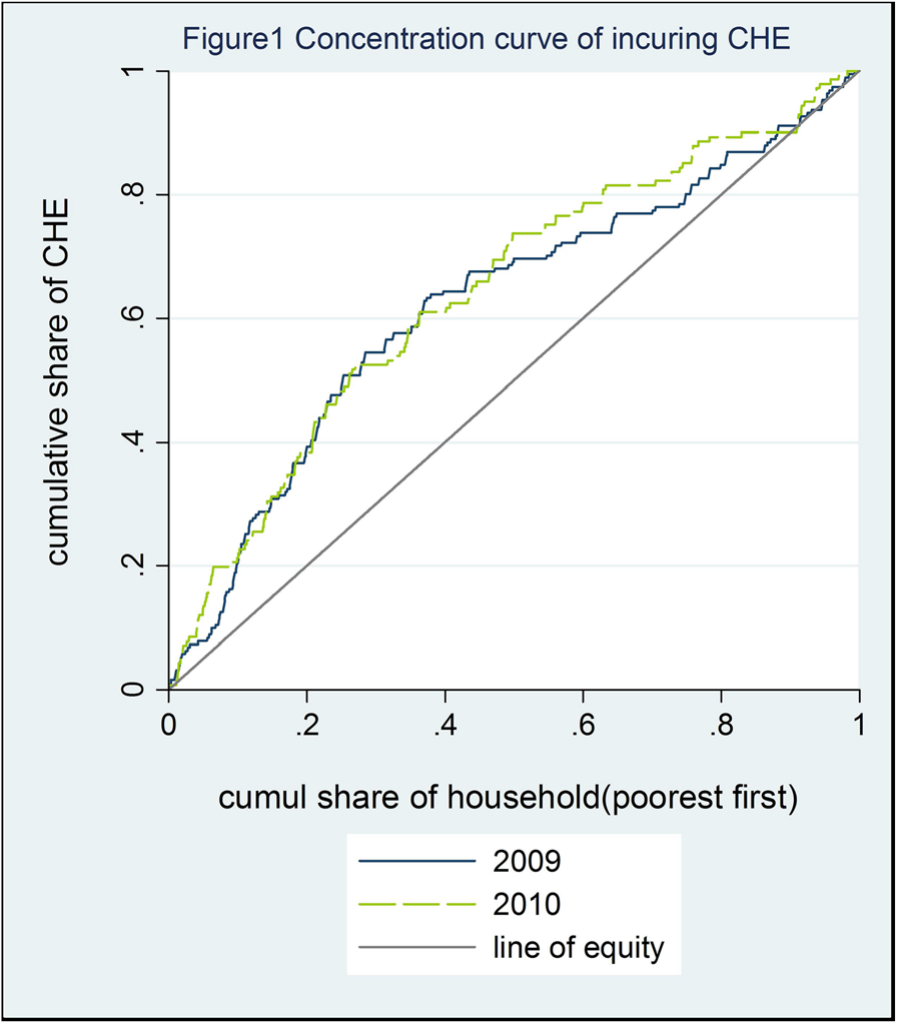
Flow chart of the sampling procedure

The statistical data were acquired from two surveys conducted in Xuzhou, the second biggest city in Jiangsu province. The first survey was conducted in 2009 and the second one was conducted in 2010. To ensure that the samples were representative of the population, a multi-stage stratified cluster random-sampling technique was used. In the first stage, Feng county and Tongshan county were sampled. In the second stage, four towns from each city were randomly sampled; thus, eight towns were sampled. In the third stage, two villages from each town were randomly sampled; thus, 16 villages were sampled. In the fourth stage, a number of families from each village, based on total population, were randomly sampled; in 2009, this sample included 1500 families. In the 2010 survey, the sample size was expanded. Besides the 1500 families, another 500 families were sampled according to the proportion of amplification; thus, a total of 2000 families were randomly sampled in 2010. After the families for which data were missing were removed from the sample, 1424 families in the 2009 data and 1796 families in the 2010 data were analyzed; the validity rate for the samples was 92%. Figure 1 illustrated the flow chart of the sampling procedure.

Data were acquired via household surveys (Additional file 1). The well-trained surveyors interviewed all members of each family according to the survey questionnaire. The survey was conducted by surveyors and survey instructors. The surveyors were responsible for performing the household survey, and the survey instructors organized, guided, and supervised the survey. The surveyors were high-level, well-trained health care personnel, and about 50 surveyors recruited from community health service centers, town health centers, and health agencies. Most surveyors were employed for both two surveys. The questionnaire used in these two surveys was the same as the one used in China’s NHSS (National Health Services Survey). The questionnaire includes general household information, individual socio-economic information, individual health conditions and health services, information of married women of childbearing age (during 15 ~ 49 years old), information of children under 5 years of age, information of elderly persons (aged 60 and above), information of diseases. This paper uses only a portion of the information gathered by the questionnaire, including the following: household income; household expenditures; and age, sex, educational qualification, use of medical services, and medical insurance status of each family member.

The Chi-square test shows that there is no statistically significant difference between this two surveys in demographic characteristics, such as household head’s gender(*χ* ^2^ = 1.87 , *p* = 0.17), age(*χ* ^2^ = 0.0031 , *p* = 0.96) and educational achievement(*χ* ^2^ = 1.75 , *p* = 0.19).

### Data calculation and statistical analyses

#### Catastrophic health expenditure

High medical expenses can throw an ordinary family into financial distress. CHE is usually used to measure the financial risk arising from the medical treatment for major diseases. If a family’s out-of-pocket (OOP) expenditure as a proportion of its capacity to pay (CTP) exceeds a specific threshold, the family is considered to experience CHE [15]. As yet, there is no consensus on the threshold value. However, based on related research literature, this paper assumes a threshold value of 40% [16]. Because the threshold value affects the measurement results and their trends, threshold values of 30 and 50% also are used, for comparative analysis. CHE is calculated as below:

$$ \mathrm{CHEi}=\left\{\begin{array}{c}1,\kern0.5em \mathrm{if}\ {OOP}_i/{CTP}_i>=0.4\\ {}0,\kern0.5em \mathrm{if}\ {OOP}_i/{CTP}_i<0.4\end{array}\right. $$

OOP refers to all self-paid expenses related to health care, including inpatient expenses, outpatient expenses, expenses for self-purchase medicine, expenses for disease prevention, and expenses for maternal and child health care (excluding the portion of these costs reimbursed by medical insurance). In this study, the OOP is calculated based on the question that how much money your family spent on the drugs, medical services and other medical products during the past year.

CTP refers to a family’s total income that is available to use as expenditures for the family’s medical care, education, and entertainment, excluding subsistence expenditure:

$$ \mathrm{CTPi}=\left\{\begin{array}{c}{Income}_i-{SE}_i,\mathrm{if}\ {SE}_i<={food}_i\\ {}{Income}_i-{food}_i, if\ {SE}_i>{food}_i\end{array}\right. $$

in which Income is the family’s total income earned during the previous year. Because the residents of developing countries are inclined to understate their actual income, some previous studies relied on total household expenditure rather than total household income. However, Chinese residents’ saving rate is as high as 51.8% [17]. If total household expenditure were used as a proxy for CTP, CTP would be underestimated and the incidence of CHE would be overestimated. Indeed, various data show that for most Chinese families total household income is greater than total household expenditure. Therefore, in this paper total household income is used to measure family CTP.

Subsistence expenditure, the minimum expenditure required for maintaining basic subsistence, is closely correlated to food expenditure. When different families in the same region have the same number of family members, their subsistence expenditures are the same regardless of household income. If a family’s food expenditure is lower than its subsistence expenditure, food expenditure can be used instead of subsistence expenditure.

Subsistence expenditure can be calculated as follows: 1) For each family, calculate food expenditure as a proportion of total household expenditure; 2) rank the families by that proportion, in ascending order; 3) select 45 to 55% of the families for use in calculating food expenditure per capita (family member); and 4) multiply food expenditure per capita by each family’s scale to calculate each family’s subsistence expenditure. Of note, given the economies of scale in family consumption, the above calculation process should use an equivalence scale rather than the actual scale for the family size [18]. The equivalence scale is calculated as follows:

$$ {\mathrm{eqsize}}_{\mathrm{i}}={\mathrm{hhsize}}_{\mathrm{i}}^{0.56} $$

in which eqsize is the scaled number of family members and hhsize is the actual number of family members.

Similarly, equivalent per-capita household income (eqinc) rather than actual per-capita household income is used to represent household income [18]:

$$ {\mathrm{eqinc}}_{\mathrm{i}}={\mathrm{i}\mathrm{ncome}}_{\mathrm{i}}/{\mathrm{eqsize}}_{\mathrm{i}} $$

### Catastrophic health expenditure inequality

The concentration index (CI) has been adopted widely as a measure of inequality in health. This paper uses the CI to measure the income-related inequality involved in CHE. The CI is defined as the area twice as large as the area between the concentration curve and the line of equality. The CI is intended to represent the extent of inequality by resorting to a summary index or, to be specific, the result of the concentration curve. The concentration index is calculated as follows:

$$ \mathrm{CI}=\frac{2}{\mu}\operatorname{cov}\left({\mathrm{CHE}}_{\mathrm{i}},{\mathrm{R}}_{\mathrm{i}}\right) $$

in which CI is the concentration index, CHE_i_ is the i^th^ family’s incidence of CHE, and R_i_ is the i^th^ family’s rank, in terms of equivalent per-capita household income, among all of the families analyzed.

The value range of the CI is [−1, 1]. When the CI is a negative value, the concentration curve is above the line of equality, indicating that CHE occurs mainly to poor families. When the CI is a positive value, the concentration curve is below the line of equality, indicating that CHE occurs mainly to rich families. The larger the absolute value of the CI is, the more severe the inequality in CHE.

### Grouping the respondents

Income-based grouping is the most commonly used method in studying health services globally [19]. By comparing various income-based groups, one can judge whether there exists a difference, or inequality, based on health status, use of health care services, and health care expenditures. The specific procedure is as follows: 1) Rank the surveyed families in ascending order in terms of equivalent per-capita household expenditure; and 2) divide the surveyed families into five income groups in ascending order (the lowest income group, the sub-low income group, the medium income group, the sub-high income group, and the highest income group).

### Multiple logistic regression

A multiple logistic regression model is adopted for analyzing the factors that influence the incidence of CHE. In the first multiple logistic regression model, the dependent variable indicates whether CHE has occurred. Independent variables in the model can be roughly classified into five types. The first type of independent variable describes the basic features of a family, for example, the number of members aged 60 years or above, whether there are any children under the age of 5 years, and family scale. The second type of independent variable describes the demographic features of the head of the family, for example, gender, age, educational level, and employment status. The third type of independent variable focuses on the family’s demand for and use of medical services, for example, whether any family member has been diagnosed with a chronic disease during the previous 6 months, whether any family member has received inpatient services during the previous year, and whether any family member has received outpatient services during the previous 2 weeks. The fourth type of independent variable focuses on medical insurance, for example, whether any family member is not covered by NCMS and whether any family member is covered by commercial insurance. The fifth type of independent variable focuses on the income-based grouping of families.

The second multiple logistic regression model in this paper is built to enable the exploration of the influence of the NCMS’s adjustment of reimbursement rates on the incidence of CHE. Because the adjustment of NCMS reimbursement rates does not affect residents that are not covered by the NCMS, the second multiple logistic regression model considers only families in which all family members are covered by the NCMS. Like the first multiple logistic regression model, the dependent variable in the second model indicates whether CHE has occurred. However, in the second model the core independent variable is the NCMS’s policy adjustment. All other independent variables from the first model are included as control variables in the second model.

All data analyses were performed using STATA 14.0 (StataCorp., College Station, Texas).

## Results

### Basic features of sample families

Summary information regarding the basic features of the sample families is shown in Table 1.The results of both surveys show that more than 75% of the families have zero or one member aged 60 years or above, more than 80% of the families have male heads, and the heads of more than 30% of the families have a junior high school education or above. In 2009, medium-sized families (those composed of three or four members) accounted for the largest proportion of the sample, 47.61%. In 2010, small families (those composed of one or two members) accounted for the largest proportion of the sample, 46.21%. Between 2009 and 2010, the proportion of families in which at least one member is a chronic-disease patient decreased from 45.08 to 43.21%, the utilization rate for inpatient services during the previous year decreased from 12.43 to 8.91%, and the utilization rate for outpatient services during the previous 2 weeks decreased from 8.22 to 3.79%. The proportion of the families in which some members are not covered by NCMS increased from 15.03 to 20.94%. (Table 1).

**Table 1:** Description of study households in 2009 and 2010 (N = number of households)

	n(%)		*χ* ^2^
Variables	2009(*N* = 1424)	2010 (*N* = 1796)	
Having elderly members			
0–1	1069 (75.07)	1373 (76.45)	0.82
≥ 2	355 (24.93)	423 (23.55)	
Having children under five			
No	1320 (92.70)	1741 (96.94)	30.43***
Yes	104 (7.30)	55 (3.06)	
Household scale			
1-2members	542 (38.06)	830 (46.21)	24.25***
3–4 members	678 (47.61)	711 (39.59)	
≥ 5 members	204 (14.33)	255 (14.20)	
Gender of household head			
Female	226 (15.87)	254 (14.14)	1.87
Male	1198 (84.13)	1542 (85.86)	
Age of household head			
Below average^a^	755 (53.02)	954 (53.12)	0.003
Over average	669 (46.98)	842 (46.88)	
Educational level household head			
Illiteracy or Elementary	978 (68.68)	1194 (66.48)	1.75
Junior high school and above	446 (31.32)	602 (33.52)	
Employment status of Household head			
Unemployment	320 (22.47)	467 (26.00)	5.36*
Employment	1104 (77.53)	1329 (74.00)	
Having chronic disease members			
No	782 (54.92)	1020 (56.79)	1.14
Yes	642 (45.08)	776 (43.21)	
Inpatient service usage^b^			
No	1247 (87.57)	1636 (91.09)	10.44***
Yes	177 (12.43)	160 (8.91)	
Outpatient service usage^c^			
No	1307 (91.78)	1728 (96.21)	28.70***
Yes	117 (8.22)	68 (3.79)	
Absence of NCMS^d^			
No	1210 (84.97)	1420 (79.06)	18.32***
Yes	214 (15.03)	376 (20.94)	
Absence of commercial health insurance^e^			
No	251 (17.63)	141 (7.85)	70.79***
Yes	1173 (82.37)	1655 (92.15)	
Income-based group			
Quintile 1 (poorest)	284 (19.94)	359 (19.99)	0.001
Quintile 2 (poorer)	285 (20.01)	359 (19.99)	
Quintile 3 (middle)	285 (20.01)	359 (19.99)	
Quintile 4 (richer)	285 (20.01)	359 (19.99)	
Quintile 5 (richest)	285 (20.01)	360 (20.04)	

* *p* < 0.05; *** *p* < 0.001

^a^ In 2009, the average age of household heads is 56.63 years old; In 2010, the average age of household heads is 58.85 years old

^b^ If anyone of the household members had used inpatient services in the last year

^c^ If anyone of the household members had used inpatient services in the last 2 weeks

^d^ If anyone of the household members did not participate in NCMS

^e^ If all household members were not covered by commercial health insurance

### Incidence of catastrophic health expenditure

The incidence of CHE varies with the choice of threshold value, as shown in Table 2. Assuming a threshold value of 30%, 240 families experienced CHE in 2009 and 211 families experienced CHE in 2010; therefore, the incidence of CHE decreased from 16.85 to 11.75% (χ^2^ = 17.190, *p* < 0.001). Assuming a threshold value of 40%, 194 families experienced CHE in 2009and 139 families experienced CHE in 2010; therefore, the incidence of CHE decreased from 13.62 to 7.74% (χ^2^ = 29.66, *p* < 0.001). Assuming a threshold value of 50%, the incidence of CHE was 10.60% in 2009 and 5.51% in 2010 (χ^2^ = 28.75, *p* < 0.001).

**Table 2:** Number and proportion of households that incurred CHE at different level of threshold value

Threshold value	2009		2010	
	n	Proportion (%)	n	Proportion (%)
30%	240	16.85	211	11.75
40%	194	13.62	139	7.74
50%	151	10.60	99	5.51

*CHE* catastrophic health care expenditure

Table 3 reports the average OOP expenditure, average CTP, and incidence of CHE among families of various income-based groups. Assuming a threshold value of 40%, the average CTP increased from 27,831 yuan (4043$)in 2009 to 41,308 yuan(6001$) in 2010, and the average OOP expenditure decreased from 3,207yuan (466$) in 2009 to 2674 yuan (388$) in 2010. In 2009, following the income-based groups from lowest income to highest income, the average OOP expenditure ranged from 1432 yuan (208$)up to 6648 yuan(966$), and the incidence of CHE ranged from 30.99% down to 8.07%. In this regard, 2010 was similar to 2009. The poorest families had the lowest OOP expenditure but experienced the highest incidence of CHE.

**Table 3:** Number and proportion of households that incurred CHE (Threshold value = 40%)

Income-based group	2009			2010		
	Average OOP health expenditure (yuan RMB)	Average household CTP (yuan RMB)	n(%) of households with CHE	Average OOP health expenditure (yuan RMB)	Average household CTP (yuan RMB)	n(%) of households with CHE
Quintile 1 (poorest)	1432.18	4592.76	88 (30.99)	1292.70	6039.48	57 (15.88)
Quintile 2 (poorer)	1927.26	11,390.38	34 (11.93)	1956.88	14,510.45	34 (9.47)
Quintile 3 (middle)	2393.90	20,093.24	25 (8.77)	2721.48	25,678.46	19 (5.29)
Quintile 4 (richer)	3626.07	30,071.82	24 (8.42)	2938.91	41,251.86	16 (4.46)
Quintile 5 (richest)	6648.21	72,925.79	23 (8.07)	4453.47	118,844.6	13 (3.61)
Total	3206.77	27,831.11	194 (13.62)	2673.68	41,308.16	139 (7.74)

*OOP* out-of-pocket, *CTP* capacity to pay

### Income related inequality in catastrophic health expenditure

Figure 1 shows the concentration curves for the incidence of CHE as of 2009 and 2010, assuming a threshold value of 40%. Both concentration curves are above the line of equality, indicating that CHE occurred mainly to poor families. The two concentration curves intersect with each other, and it is impossible to judge the extent of inequality and which concentration curve is farther from the line of equality. As shown in Table 4, the CI of CHE was −0.298 (95% confidence interval: −0.377 to −0.221) in 2009 and −0.323 (95% confidence interval: −0.413 to −0.233) in 2010. The CI was negative in both 2009 and 2010, indicating that CHE occurred mainly to poor families. This CI index result is consistent with the result indicated by the concentration curves. The absolute value of the 2010 CI was greater than the absolute value of the 2009 CI.

**Table 4:** Concentration index of CHE

Threshold value	2009		2010	
	Concentration index	95%CI	Concentration index	95% CI
30%	−0.321	(−0.387,-0.255)	−0.362	(−0.429,-0.294)
40%	−0.298	(−0.377,-0.221)	−0.323	(−0.413,-0.233)
50%	−0.284	(−0.378,-0.189)	−0.318	(−0.426,-0.210)

### Factors influencing the incidence of catastrophic health expenditure

According to the estimation of the first multiple logistic regression model, NCMS coverage is statistically significantly correlated to the incidence of CHE. Compared with rural families in which all members are covered by the NCMS, rural families in which some members are not covered by the NCMS have an ORm(the odds ratio of multivariate logistic regression analysis) of 0.65 for CHE (95% confidence interval: 0.43 to 1.00). Another important independent variable is household income. Compared with the poorest families, families with greater income experience significantly lower incidence of CHE (p<0.001). (Table 5).

**Table 5:** Factors associated with the occurrence of CHE

Variables	n(%)	ORu^a^ (95%CI)	ORm^b^ (95%CI)
Having elderly members			
0–1	169 (6.92)	1	1
≥ 2	164 (21.08)	3.59 (2.85,4.53)^***^	1.32 (0.96,1.82)
Having children under five			
No	320 (10.45)	1	1
Yes	13 (8.18)	0.76 (0.43,1.36)	1.32 (0.64,2.73)
Household scale			
1-2members	192 (13.99)	1	1
3–4 members	111 (7.99)	0.53 (0.42,0.68)^***^	0.88 (0.65,1.18)
≥ 5 members	30 (6.54)	0.43 (0.29,0.64)^***^	0.45 (0.27,0.74)^**^
Gender of household head			
Female	80 (16.67)	1	1
Male	253 (9.23)	0.51 (0.39,0.67)^***^	0.84 (0.60,1.19)
Age of household head			
Below average	82 (4.80)	1	1
Above average	251 (16.61)	3.95 (3.05,5.13)^***^	1.52 (1.02,2.25)^*^
Educational level of household head			
Illiteracy or elementary	278 (12.80)	1	1
Junior high school and above	55 (5.25)	0.38 (0.28,0.51)^***^	0.87 (0.61,1.25)
Employment status of household head			
Unemployment	167 (21.22)	1	1
Employment	166 (6.82)	0.27 (0.22,0.34)^***^	0.61 (0.45,0.82)^**^
Having chronic disease members			
No	84 (4.66)	1	1
Yes	249 (17.56)	4.36 (3.36,5.64)^***^	2.17 (1.62,2.92)^***^
Inpatient service usage			
No	198 (6.87)	1	1
Yes	135 (40.06)	9.06 (6.98,11.77)^***^	10.48 (7.62,14.41)^***^
Outpatient service usage			
No	308 (10.15)	1	1
Yes	25 (13.51)	1.38 (0.89,2.14)	0.97 (0.57,1.64)
Absence of NCMS			
No	301 (11.44)	1	1
Yes	32 (5.42)	0.44 (0.30,0.65)^***^	0.65 (0.43,1.00)^*^
Absence of commercial health insurance			
No	19 (4.85)	1	1
Yes	314 (11.10)	2.45 (1.52,3.95)^***^	1.19 (0.68,2.09)
Income-based group			
Quintile1 (poorest)	145 (22.55)	1	1
Quintile 2 (poorer)	68 (10.56)	0.41 (0.30,0.55)^***^	0.53 (0.37,0.76)^**^
Quintile 3 (middle)	44 (6.83)	0.25 (0.18,0.36)^***^	0.42 (0.27,0.64)^***^
Quintile 4 (richer)	40 (6.21)	0.23 (0.16,0.33)^***^	0.39 (0.25,0.62)^***^
Quintile 5 (richest)	36 (5.58)	0.20 (0.14,0.30)^***^	0.35 (0.22,0.56)^***^

A total of 3220 cases were include in the multivariate logistic regression

* *p* < 0.05; ** *p* < 0.01; *** *p* < 0.001

^a^ ORu: the odds ratio of univariate logistic regression analysis

^b^ ORm: the odds ratio of multivariate logistic regression analysis

The incidence of CHE is correlated to a variety of factors, including family scale, family head’s age, family head’s employment status, presence of a chronic-disease patient in the family, family members’ use of inpatient services during the previous year, family members’ NCMS coverage, and household income (refer to Table 5). In contrast, the incidence of CHE is not correlated to the number of aged family members, the number of children under five, the family head’s gender, the family head’s educational level, family members’ use of outpatient services, and family members’ commercial insurance coverage. The ORm for CHE of large families compared with small families (those composed of one to two members) is 0.45 (*p* = 0.002). The incidence of CHE is higher for families with older family heads than for families with younger heads: ORm is 1.52 (*p* = 0.039). The incidence of CHE is lower for families in which the head is employed than for families in which the head is unemployed: the ORm is 0.61 (*p* = 0.001). The incidence of CHE is elevated by the presence of a chronic-disease patient in the family: the ORm is 2.17 (*p* < 0.001). The incidence of CHE is higher for families in which a member has accessed inpatient services during the previous year: the ORm is 2.17 (*p* < 0.001).

Table 6 reports the estimated influences of the adjustment of NCMS reimbursement policy on CHE. The second multiple logistic regression model incorporates a new variable (adjustment of NCMS reimbursement policy) for this purpose, and is estimated based only on data for families in which all members are covered by the NCMS. Controlling for other factors, the incidence of CHE is lower (ORm = 0.48, *p* < 0.001) subsequent to adjustment of reimbursement policy. The estimated results for other independent variables are similar to those in the first multiple logistic regression model. The incidence of CHE is significantly associated with a variety of factors, including family scale, family head’s age, family head’s employment status, presence of a chronic-disease patient in the family, family members’ use of inpatient services, and household income.

**Table 6:** Impact of NCMS adjustment on CHE occurrence

Variables	n(%)	ORu^a^(95%CI)	ORm^b^(95%CI)
NCMS adjustment			
Before	181 (14.96)	1	1
After	120 (8.45)	0.52 (0.41,0.67)^***^	0.48 (0.36,0.64)^***^
Having elderly members			
0–1	143 (7.30)	1	1
≥ 2	158 (23.58)	3.92 (3.06,5.02)^***^	1.39 (0.99,1.96)
Having children under five			
No	294 (11.60)	1	1
Yes	7 (7.29)	0.60 (0.27,1.31)	0.91 (0.35,2.38)
Household scale			
1-2members	177 (14.74)	1	1
3–4 members	101 (8.99)	0.57 (0.44,0.74)^***^	0.78 (0.56,1.08)
≥ 5 members	23 (7.54)	0.47 (0.30,0.74)^**^	0.44 (0.25,0.79)^**^
Gender of household head			
Female	73 (19.06)	1	1
Male	228 (10.15)	0.48 (0.36,0.64)^***^	0.85 (0.58,1.24)
Age of household head			
Below average	66 (4.91)	1	1
Above average	235 (18.27)	4.33 (3.26,5.76)^***^	1.58 (1.03,2.41)^*^
Educational level of household head			
Illiteracy or Elementary	253 (14.00)	1	1
Junior high school and above	48 (5.83)	0.38 (0.28,0.52)^***^	0.98 (0.66,1.45)
Employment status of household head			
Unemployment	149 (23.54)	1	1
Employment	152 (7.61)	0.27 (0.21,0.34)^***^	0.62 (0.45,0.85)^**^
Having chronic disease members			
No	72 (4.92)	1	1
Yes	229 (19.64)	4.73 (3.58,6.24)^***^	2.20 (1.60,3.02)^***^
Inpatient service usage			
No	178 (7.59)	1	1
Yes	123 (43.16)	9.24 (6.99,12.23)^***^	10.95 (7.77,15.45)^***^
Outpatient service usage			
No	280 (11.30)	1	1
Yes	21 (13.73)	1.25 (0.77,2.01)	0.82 (0.46,1.46)
Absence of commercial health insurance			
No	14 (5.24)	1	1
Yes	287 (12.15)	2.50 (1.44,4.34)^**^	1.43 (0.74,2.79)
Income-based group			
Quintile1 (poorest)	139 (23.76)	1	1
Quintile 2 (poorer)	64 (11.76)	0.43 (0.31,0.59)^***^	0.59 (0.40,0.86)^**^
Quintile 3 (middle)	37 (7.13)	0.25 (0.17,0.36)^***^	0.46 (0.29,0.73)^**^
Quintile 4 (richer)	35 (6.97)	0.24 (0.16,0.36)^***^	0.41 (0.25,0.67)^**^
Quintile 5 (richest)	26 (5.42)	0.18 (0.12,0.28)^***^	0.31 (0.18,0.54)^***^

A total of 2630 were included in the multivariate logistic regression

* *p* < 0.05; ** *p* < 0.01; *** *p* < 0.001

^a^ ORu: the odds ratio of univariate logistic regression analysis

^b^ ORm: the odds ratio of multivariate logistic regression analysis

## Discussion

### The incidence of catastrophic health expenditure is somewhat reduced but still extremely high among poor families

In this paper, an index of CHE is used to measure the financial risks of health care among rural residents of Jiangsu Province. Among the surveyed families, the incidence of CHE decreased from 13.62% in 2009 to 7.74% in 2010. On the whole, the financial risks of health care among rural families decreased with the rise in the NCMS reimbursement rates. However, the incidence of CHE was still extremely high, and CHE occurred mainly to the poorest families. Previous studies pointed out that the low-income families were always apt to pay a somewhat high ratio of OOP relative to their household incomes [20, 21]. Su believed that low-income families always experience higher incidence of CHE than rich families, regardless of the threshold value used in defining CHE [22].

Research also has shown that low-income groups suppress their demand for medical services. Superficially, the poorest families have the lowest OOP expenditure, less than half of the average OOP expenditure. Perhaps this is not because they have good health and therefore low demand for medical services, but rather because, due to limited financial capacity, they suppress their demand for medical services. For low-income families, even a very small amount of medical expenditure can be catastrophic [22]. In 2009 and 2010, the poorest families had the highest incidence of CHE. This result implies that, for financial reasons, some poor families’ health need is not converted into effective demand, that is to say, some poor patients cannot afford to see a doctor.

Poor rural families lack the capacity to pay for medical expenses and therefore are more vulnerable to the huge financial effects of high medical expenses [23]. Therefore, it is imperative that governments should take measures to make medical services more accessible to poor and low-income rural families and to help them withstand the financial risks of various diseases.

### Socioeconomic inequality in catastrophic health expenditure has been somewhat aggravated

In this paper, the concentration curve and CI are used to measure socioeconomic inequality in CHE. They show that there exists a pro-rich inequality in financial risk protection, that is to say, CHE occurs mainly to poor families. By using regression analysis to examine the factors that influence CHE, this paper finds that household income significantly influences the incidence of CHE. According to the regression results, households with higher income have lower probability of CHE, which is consistent with the CI-based conclusion.

Between 2009 and 2010, the income-related inequality was somewhat aggravated, unexpectedly, despite the decline in the overall incidence of CHE. The rise in the NCMS reimbursement rates failed to reduce the income-related inequality in CHE. Perhaps it is because the rise in the NCMS reimbursement rates favored high-income families. Due to certain binding clauses in the NCMS’s policy, the NCMS cannot exert its full influence on poor families. For example, the NCMS can provide reimbursement only for OOP expenditures above the baseline deductibles [24]; however, even OOP expenditures below the deductibles are a heavy financial burden for the poorest families. Meanwhile, NCMS imposed cap line (limit amount money) on reimbursement (80,000 Yuan, equal to 11,613 $), thus the patients had to pay the medical fee exceeding the cap line by themselves. If the amount of medical expenditure was very large, the copayment of the patients would be great. For rich families, the cap amount could effectively reduce the disease burden although the compensation was limited. For poor families, however, the copayment could still be unaffordable and might use up the family disposable income, or widely spend. Hence, the copayment more likely means CHE for poor families than rich families, even if covered by NCMS. On the other words, so far, the improvement of NCMS played a greater role to reduce disease burden for rich families than it did for poor families.

Although the incidence of CHE was reduced, on the whole, the aggravation of income-related inequality offset or at least mitigated the positive effect.

### Increased reimbursement rates reduce the incidence of catastrophic health expenditure

Research has not yet provided evidence to support the view that the NCMS can reduce the incidence of CHE. Compared with families in which all members are covered by the NCMS, however, families in which some members are not covered by the NCMS have a lower incidence of CHE. One possible reason is that NCMS coverage causes families to use medical services more frequently than they otherwise would. Low-income families’ demand for medical services is highly elastic [14]. The rise in the access rate for medical services outweighs the decline in the prices of those medical services. As a result, the OOP expenditure increases and, therefore, the probability of CHE rises. This finding is the same as the conclusion drawn by Wagstaff [14] and Ekman [25].

Another possible reason for the lack of conclusive evidence for the NCMS reducing the incidence of CHE is adverse selection. In medical insurance, adverse selection means that, given the same insurance premium and insurance participation based on voluntary choice, high-risk people are more inclined to buy medical insurance but low-risk people are more inclined not to buy medical insurance [26]. If a family has some members who have high health risk, the family is more likely than other families to participate in the NCMS and even to have all of its members participate in the NCMS. However, such a family also is more likely to experience CHE.

Based on the results of the second multiple logistic regression model, if all of the members of a family participate in the NCMS, the rise in the NCMS reimbursement rates significantly reduces the incidence of CHE, when controlling for other independent variables. The rise in the NCMS reimbursement rates is equivalent to a further decline in the prices of medical services, thus helping to reduce medical expenditure. To some extent, the rise in the NCMS reimbursement rates plays a positive role in reducing the rural families’ financial burden of medical services.

Meanwhile, families that include chronic-disease patients and families in which a member has accessed inpatient services during the previous year had relatively high incidence of CHE. In contrast, large families and high-income families had relatively low incidence of CHE. Being with chronic diseases fit the common features of CHE, as well as inpatient services utilization. Generally, families with more members meant with higher family income, and the probability of incidence of CHE was relatively smaller. Hence, CHE more likely occurred among the low-income families with chronic disease patients who needed to use long-term inpatient healthcare.

### Implications for policy and practice

Today, the actual reimbursement rates for China’s basic medical insurance system, including the NCMS, are still very low [27], and rural families face a heavy disease burden. To reduce the incidence of CHE, it is necessary to increase the NCMS financing capacity, step by step, and raise the reimbursement rates for medical expenditures, especially for inpatient expenditures. Meanwhile, it is also necessary to strengthen the overall arrangement of medical insurance funds, thus enlarging the pool of medical insurance funds and improving medical insurance’s ability to reduce risk.

Major diseases are an important factor that gives rise to CHE [28]. To help rural families withstand the financial risks arising from major diseases, governments should develop critical illness insurance systems, which reimburse families for the high medical expenses associated with major diseases, or critical illness assistance systems, which provide financial relief to families that experience CHE.

To some extent, the medical insurance system’s unreasonable reimbursement policy suppresses low-income people’s demand for medical services. Rural families covered by the NCMS usually access medical services on a “first-pay and then reimburse” basis [29]. Some low-income families may be forced to forgo medical treatment, because they cannot afford to pay before being reimbursed. Therefore, an instant reimbursement policy or prepayment system should be implemented as part of the reform of the medical insurance system. When insured people, reimburse them for the medical expenses instantly so that they only need pay the OOP expenditure [15]. Furthermore governments and insurance institutions could prepay the entire medical expenses temporarily. Such an instant reimbursement policy or prepayment system could unleash at least some of the suppressed demand for medical services and, particularly, help reduce the disease financial burden for poor and low-income rural families and enable more rural patients to receive timely medical treatment.

Currently, the distinctive policies for families with different income levels are unachievable in the basic medical insurance system including NCMS. Then, in the future, it is very necessary to develop policies targeted on poor families.

Additionally, the possibility of CHE incidence was relatively large among the disadvantageous families with the features of low income, with chronic disease patients who needed long-term inpatient services. Policy makers should pay more attention to these rural families with the above characteristics than other populations.

### Limitation

There are some limitations in the study. Firstly, there is a problem about representativeness of the sample. In this study, the sample only included participants from city Xuzhou and conclusions cannot be generalized to the entire population in China. Secondly, some potential relevant factors, such as the medical service utilization and the level of healthcare institutions, have not been considered in the study. The low-income groups tend to use primary-level care, and this may due to the lower medical expenses comparing to high-income groups. In this case, this may slightly influence the CHE.

## Conclusions

Based on the 2009 and 2010 surveys of rural families in two counties of Xuzhou, the overall incidence of CHE somewhat decreased during that period, but income related inequality in CHE was aggravated and the incidence of CHE tended to increase among poor families. The rise in the NCMS reimbursement rate significantly reduced the incidence of CHE among the rural families covered by NCMS. With the constantly increase of the NCMS coverage (It is currently close to a universal coverage for NCMS and other basic social health insurance in China [30]), the impact of reimbursement rate rising should get an extension.

## Additional file

Additional file 1: Major variables and related survey questions. (DOCX 19 kb)

## Abbreviations

CHE: Catastrophic health expenditure
CTP: Capacity to pay
NCMS: New Rural Cooperative Medical System
NHSS: National Health Service Survey
OOP: Out-of-pocket
