# Supplementary material for: Income related inequality and influencing factors: a study for the incidence of catastrophic health expenditure in rural China
Source: BMC Public Health. 2017 Sep 20;17:727. doi: 10.1186/s12889-017-4713-x (PMC5607576; doi:10.1186/s12889-017-4713-x)
Supplement: Additional file 1: — Major variables and related survey questions. (DOCX 19 kb) [file 12889_2017_4713_MOESM1_ESM.docx]

Major variables and related survey questions

1. Variable: CHE

CHE is calculated based on income, out-of-pocket expenses and food expenses

Question:

What is your family’s annual net income (Yuan) the year before?

How much did your family spend on medicines, medical services and supplies the year before (after reimbursement)?

How much did your family spend on food the year before?

1. Variables: Having elderly members, Having children under five，Age of household head

These three variables are calculated based on the birth year.

Question:

What’s your birth year?

1. Variable: Household scale

Question:

How many people are there in your family(living together)?

1. Variable: Gender of household head

Question:

Gender (1) Male (2) Female

1. Variable: Educational level household head

Question:

What’s your educational achievement? (1) Illiteracy (2) Elementary school (3) Middle school (4) High school / Technical school (5) Technical secondary school / vocational school (6) Junior college (7) University and above

1. Variable: Employment status of Household head

Question:

What’s your employment status? (1) Employed (2) Retirement (3) School student (4) unemployed

1. Variable: Having chronic disease members

Question:

Do you have chronic disease diagnosed by doctor in the recent six month？ (1) Yes (2) No

1. Variable: Inpatient service usage

Question:

Did your use inpatient services in the last year? (1) Yes (2) No

1. Variable: Outpatient service usage

Question:

Did you use outpatient services in the last two weeks? (1) Yes (2) No

1. Variable: Absence of NCMS

Question:

Do you take part in the NCMS? (1) Yes, I do (2) No, I don’t (3) I used to do but now I have exited

1. Absence of commercial health insurance

Question:

Do you buy commercial insurance? (1) Yes (2) No

1. Variable: Income-based group

This variable is calculated based on family income and household scale.

Question:

What is your family’s annual net income (Yuan) the year before?

How many people are there in your family(living together)?
